# The electrophysiological characteristics of social exclusion: the perspective of close and distant relationships

**DOI:** 10.3389/fpsyg.2023.1010493

**Published:** 2023-04-27

**Authors:** Pengcheng Zhang, Min Zhu, Jingjing Hu, Xiangping Gao

**Affiliations:** ^1^Department of Psychology, School of Education, Zhejiang International Studies University, Hangzhou, China; ^2^Department of Social Work and Management, Nanjing Tech University, Nanjing, China; ^3^Academic Affairs Office, Shanghai Normal University, Shanghai, China

**Keywords:** social exclusion, close and distant relationships, electrophysiological characteristics, ERP, static passing ball paradigm

## Abstract

The sources of social exclusion are very wide, ranging from the closest people to strangers. However, current studies mainly reveal the electrophysiological characteristics of social exclusion by means of binary comparison between social exclusion and social inclusion, and lack of in-depth analysis of the differences caused by different sources of exclusion. In this study, a static passing ball paradigm system including close and distant relationship identity information was used to reveal the electrophysiological characteristics of individuals when they were excluded by people with different close and distant relationships. The results showed that there was a degree effect of P2, P3a, and LPC components when individuals were excluded by people with different close and distant relationships. Specifically, the amplitude of P2, P3a, and LPC components was larger when individuals were excluded by more distant people. The results indicated that individuals would become more alert and perceive stronger exclusion experience when they were excluded by more distant people, which provided more diversified evidence for the conclusion that electrophysiological components were larger under the condition of exclusion, and revealed the electrophysiological basis behind the multiple motivation models. The results also helped to explain the physiological reasons behind individuals’ different coping behaviors toward excluder with different importance of relationship.

## 1. Introduction

Social exclusion refers to the phenomenon and process in which person’s belonging needs and relationship needs are hindered due to being excluded by a social group or others (Williams, 2007, 2009). The temporal need-threat model (Williams, 2007, 2009; Ren et al., 2018) pointed out that the influence of social exclusion on individuals can be divided into three stages over time: reflexive stage, reflective stage, and resignation stage. The reflexive stage causes social pain in individuals and results in significantly reduced satisfaction with belonging, self-esteem, control, and meaningful presence. During the reflective stage, individuals typically respond with antisocial, prosocial, or avoidant behaviors in an attempt to recover from the negative effects of social exclusion. During the resignation stage, individuals suffer from feelings of alienation, depression, helplessness, and worthlessness due to long-term exclusion.

The neurophysiological basis behind the above-mentioned external manifestations caused by social exclusion has been extensively and deeply studied by researchers using functional magnetic resonance imaging (fMRI) technology in the past. On the basis of a large number of studies, some researchers have conducted a review and meta-analysis of this research in recent years. For example, Mwilambwe-Tshilobo and Spreng (2021) found, through a meta-analysis of social exclusion studies using the Cyberball paradigm, the ventral anterior cingulate cortex (vACC) and posterior cingulate cortex (PCC), inferior frontal gyrus and superior frontal gyrus (IFG and SFG), posterior insula, and occipital pole showed activation responses. Wang et al. (2017) summarized the following brain regions closely related to social exclusion through comparative analysis: anterior cingulate cortex (ACC), posterior cingulate cortex (PCC), ventral prefrontal cortex (vPFC), ventrolateral prefrontal cortex (vlPFC), and insula and temporal lobe.

According to the above studies, the brain regions activated when an individual was excluded mainly involve the anterior and posterior cingulate cortex, insula, and prefrontal regions. In addition to neuroimaging studies, some researchers have also explored the electrophysiological characteristics of social exclusion using event-related potential (ERP) techniques. For example, Baddam et al. (2016) found that stranger exclusion induced greater prefrontal P2 component amplitudes compared to friend exclusion. In addition, studies have shown that social exclusion condition induces larger amplitudes of the fronto-parietal N2 component than social inclusion condition (Themanson et al., 2013; Weschke and Niedeggen, 2013). Another study using the passing ball paradigm showed that in the relatively early stage of 300–400 ms, social exclusion would induce negative emotions in the participants, manifesting as a larger amplitude of the induced P3a component (Gutz et al., 2011; Weschke and Niedeggen, 2013).

Following the P3a component, social exclusion induces the P3b component, which is involved in the attention, evaluation, and classification of stimuli (Kiat et al., 2017; Kiat and Cheadle, 2017). For example, Kiat et al. (2018) found that the social exclusion group evoked larger amplitude of the P3b component, indicating that individuals paid more expected attention during the exclusion period. The validity of the P3b component as an electrophysiological marker for distinguishing between social exclusion and social inclusion under the passing ball paradigm has been repeatedly demonstrated. For example, some studies have shown that the P3b component evoked in social exclusion conditions has greater amplitudes than in social inclusion conditions (Crowley et al., 2010; Themanson et al., 2013; Weschke and Niedeggen, 2013, 2016; Gutz et al., 2015). However, there are also some studies that have reached the opposite conclusion: the P3b component induced by social inclusion conditions is larger than that of social exclusion conditions (Gutz et al., 2011; Weschke and Niedeggen, 2015).

According to the above studies, in terms of the neurophysiological basis, most of the studies use the traditional passing ball paradigm to conduct a binary comparative analysis of social exclusion and social inclusion. The shortcoming of research of Baddam et al. (2016) is that strangers and friends are put together for passing ball game interaction, so it fails to completely distinguish friend exclusion from stranger exclusion. The multiple motivation model (Smart Richman and Leary, 2009) pointed out that individuals will adopt different behaviors to deal with different close and distant relationships excluders. For example, if an individual perceives a relationship with an excluder as very important (such as with a parent), they may be inclined to try to repair the relationship and exhibit prosocial behavior. Conversely, they are more likely to exhibit antisocial or avoidance behavior. That is, close and distant relationships can regulate the coping behavior of individuals after being excluded (Zhang et al., 2023). Furthermore, Nezlek et al. (2012) found that 32% of individual exclusion came from strangers, 30% from unfamiliar people, 16% from ordinary friends, 13% from close friends, 4% from partners, and 5% from relatives. The extensiveness of the sources of social exclusion also illustrates the need to study the moderating effects caused by differences in the sources of exclusion. Close and distant relationships can regulate coping behavior. What is the neurophysiological basis behind differential coping behavior? This is a question that has not been answered by previous dichotomous comparative studies.

Therefore, this study will conduct a deep analysis of the differences caused by different sources of exclusion, and try to clarify the electrophysiological characteristics of individuals when they are excluded by people with different close and distant relationships, reveal the neurophysiological basis behind different behavioral choices, and help understand the physiological reasons behind making a behavioral choice. In addition, as mentioned above, the research conclusions on the electrophysiological characteristics of social exclusion are not uniform, and the discussion of this issue from the perspective of close and distant relationships will also provide more diversified evidence for the qualitative description of electrophysiological characteristics.

To explore the research questions raised, college students will be selected as participants in this research and conducted in the following form. First, referring to the classification in the paper of Nezlek et al. (2012), parents were selected as the objects closest to the individual, strangers as the objects with the furthest relationship with the individual, and friends whose relationship in the middle of parents and strangers was written by the participants were regarded as objects with medium relationship, thus forming three groups of close and distant exclusion experimental groups with obvious gradient difference (parent exclusion, friend exclusion, and stranger exclusion). In addition, the situation of acceptance by all others except oneself was used as a common control group, named as other-inclusion. Secondly, many researchers add names or avatars next to the virtual figures when using the dynamic ball passing paradigm of three-or four-person interaction to help participants better identifying information and enhance the ecological validity of the experiment. For example, one virtual figure is of the participant himself, and the other two are of strangers. Except for the participant’s name or avatar, the names or avatars next to the other two strangers remain the same in the exclusion group and the inclusion group. Different from previous studies, the identity information of the exclusion group and the inclusion group in this study, in addition to the participants, the identities of the other two interacting people will also change. For example, the parent exclusion group is interacting with parents, and the friend exclusion group is interacting with friends. Therefore, if the method of name or avatar is adopted, the names or avatars next to the people interacting with the participants in the four groups of this study cannot remain unchanged, thus adding an additional variable. In order to enhance the control of this additional variable, the color-identity association paradigm is used to connect virtual figurines representing different identities with the same color, which could well ensure the consistency of the information of the four groups except the different identity information. Finally, the ball is thrown from one player to another during player interaction in the traditional dynamic ball passing paradigm, and the movement of the ball during this process also induces electrophysiological components (Smith et al., 2008), which appear at the same time as the electrophysiological components induced by the exclusion, thus forming interference. To overcome this deficiency, this study will use the static passing ball paradigm to initiate social exclusion and social inclusion, drawing on the research of Ikeda and Takeda (2019). The specific operation methods are as follows: the other two players are always passing the ball to each other, and the ball is rarely passed to the participant to initiate social exclusion, the probability of passing the ball and not passing the ball to the participant is equal to initiate social inclusion.

## 2. Methods

### 2.1. Participants

A total of 96 college students (45 males) were recruited through campus advertisements and randomly assigned to four groups. After the experiment, it was found that one student had incomplete data records, two students misunderstood the instruction, resulting in too many key press errors, and the quality of EEG data recorded by four students was too poor, so the data of these seven students were excluded. Finally, there were 20 participants in the other inclusion group (M_age_ = 19.45 ± 1.10 years, eight males), 23 participants in the parent exclusion group (M_age_ = 19.22 ± 0.90 years, 12 males), 24 participants in the friend exclusion group (M_age_ = 19.21 ± 0.83 years, 12 males), and 22 participants in the stranger exclusion group (M_age_ = 18.86 ± 0.83 years, 11 males). The participants were all right-handed, healthy, no color blindness or color weakness, no history of brain injury or neurological disease, and normal or corrected vision. Before the start of the experiment, the participants were asked to fill in the basic information and read and sign the informed consent form of the experiment. After the experiment was completed, they would be paid accordingly. This research was approved by the Academic Ethics and Ethics Committee of the corresponding author’s University.

### 2.2. Research design

A single-factor between-subject experimental design was adopted, and the independent variable was the social priming group (four levels: parent exclusion, friend exclusion, stranger exclusion, and others inclusion). The dependent variable was the corresponding electrophysiological component evoked by the stimulus.

### 2.3. Measuring tools

#### 2.3.1. IOS questionnaire

The Inclusion of Others in Self (IOS) questionnaire compiled by Aron et al. (1992) was used to measure the intimacy between participants and others. The IOS questionnaire consists of seven double circles with linearly increasing overlap to form a seven-point isometric scale. The retest reliability of this questionnaire is 0.83. In recent years, some researchers have further proved the reliability of this questionnaire (Gächter et al., 2015).

#### 2.3.2. Post-cyberball questionnaire

The Post-cyberball questionnaire compiled by Williams (2009) was used to measure the priming effect of the social exclusion experiment. The questionnaire included four sub-dimensions: belonging, self-esteem, sense of control, and sense of meaning. Each sub dimension contains five questions, for a total of 20 questions. The questionnaire uses a seven-point scoring method to ask participants to judge the degree of agreement with the description of the topic, ranging from completely disagree to completely agree. In this study, the four sub dimensions of total questionnaire (*α* = 0.94), belonging (*α* = 0.85), self-esteem (*α* = 0.79), sense of control (*α* = 0.81), and sense of meaning (*α* = 0.88) all showed good internal consistency.

### 2.4. Procedures and materials

First, the participants were asked to complete the IOS scale through the instruction, and the participants were asked to judge the intimacy between him and his parents and strangers. Secondly, let him write the names of two friends whose intimacy with him is in the middle of parents and strangers. Then, the experiment was carried out in two stages. In the first stage, participants were asked to connect different colored figurines with different identities so as to increase the sense of substitution and ecological validity of the subsequent experiments. The second stage was to explore the electrophysiological characteristics of individuals when they were excluded by people with different close and distant relationships.

#### 2.4.1. Stage 1: color-identity connection task

**Figure 1:**
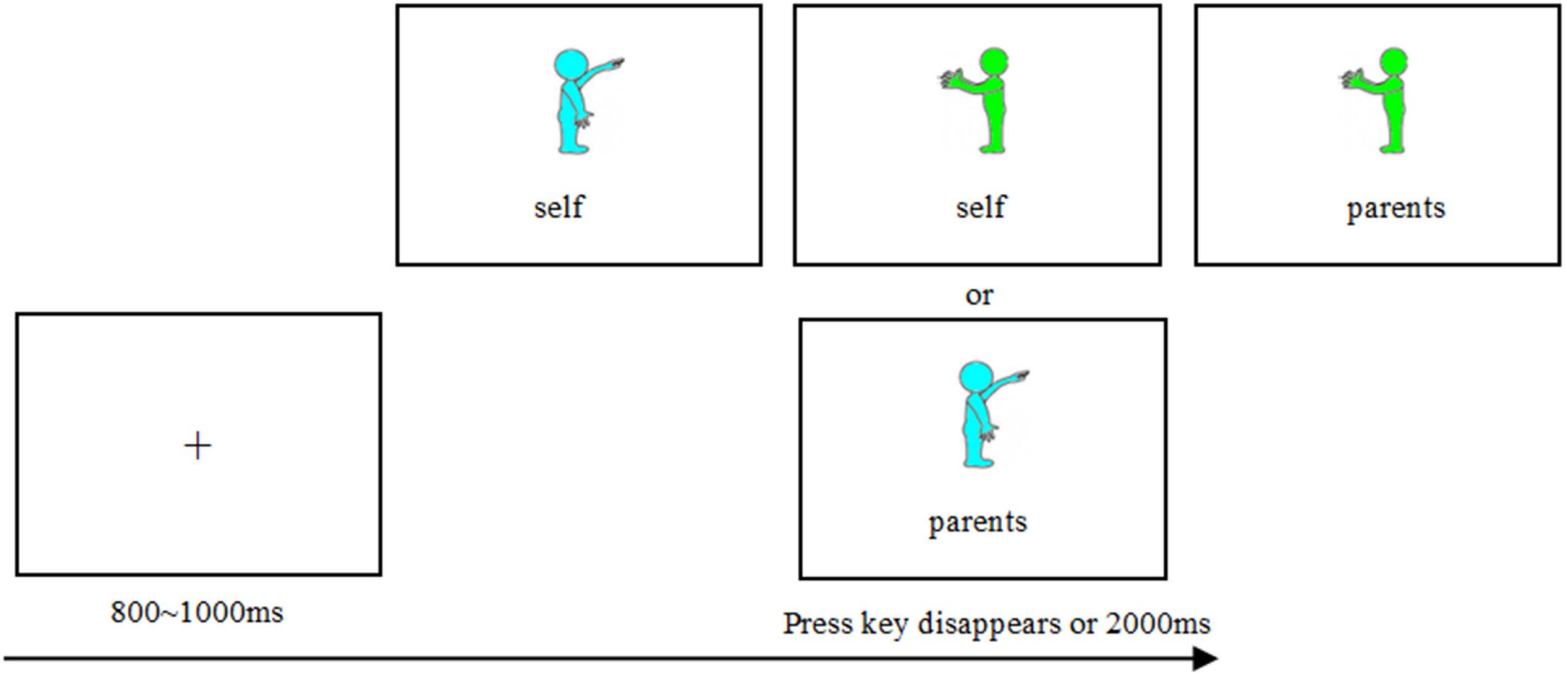
Schematic diagram of the color-identity connection task experiment process.

The participants were assigned different colored figurines (see Figure 1) to represent different close and distant relationships people through instructions. Since it was an inter-group experiment, the cyan figures represented parents, friends, strangers, and others in different groups, while the green figures always represented the participants, and the colors were balanced among the participants. The specific process of each trial (see Figure 1) was as follows: ①The gaze point “+” appears in the center of the screen to remind the participants to concentrate. ②At the same time, different colored figurines and identity naming were presented. The task of the participants was to judge whether the colored figurines were consistent with the identity naming. If there were consistent, press the “F” key; if there were inconsistent, press the “J” key. The keys were balanced among the participants.

Participants first completed 12 trials of exercises (three times for each level in the Figure 1) to familiarize themselves with the experimental process and operation. When the accuracy rate reached 95% or more, they entered the formal experiment. The procedure of each trial in the formal experiment was the same as in the exercise phase. Each level was presented 12 times, for a total of 48 trials. A large number of studies have proved that this method can achieve a good connection between colored figurines and identity (Sui et al., 2012, 2013).

#### 2.4.2. Stage 2: passing ball task

Drawing lessons from previous studies (Zadro et al., 2004; Ikeda and Takeda, 2019), the participants were told through instructions that this experiment will train their mental visualization skills in experimental games, and they need to use their mental imagination according to the situation presented in the experiment. During the experiment, static pictures were used to simulate the dynamic passing ball process, and the participants were required to immerse their imagination and judge whether parent/friend/stranger/others passed the ball to them.

**Figure 2:**
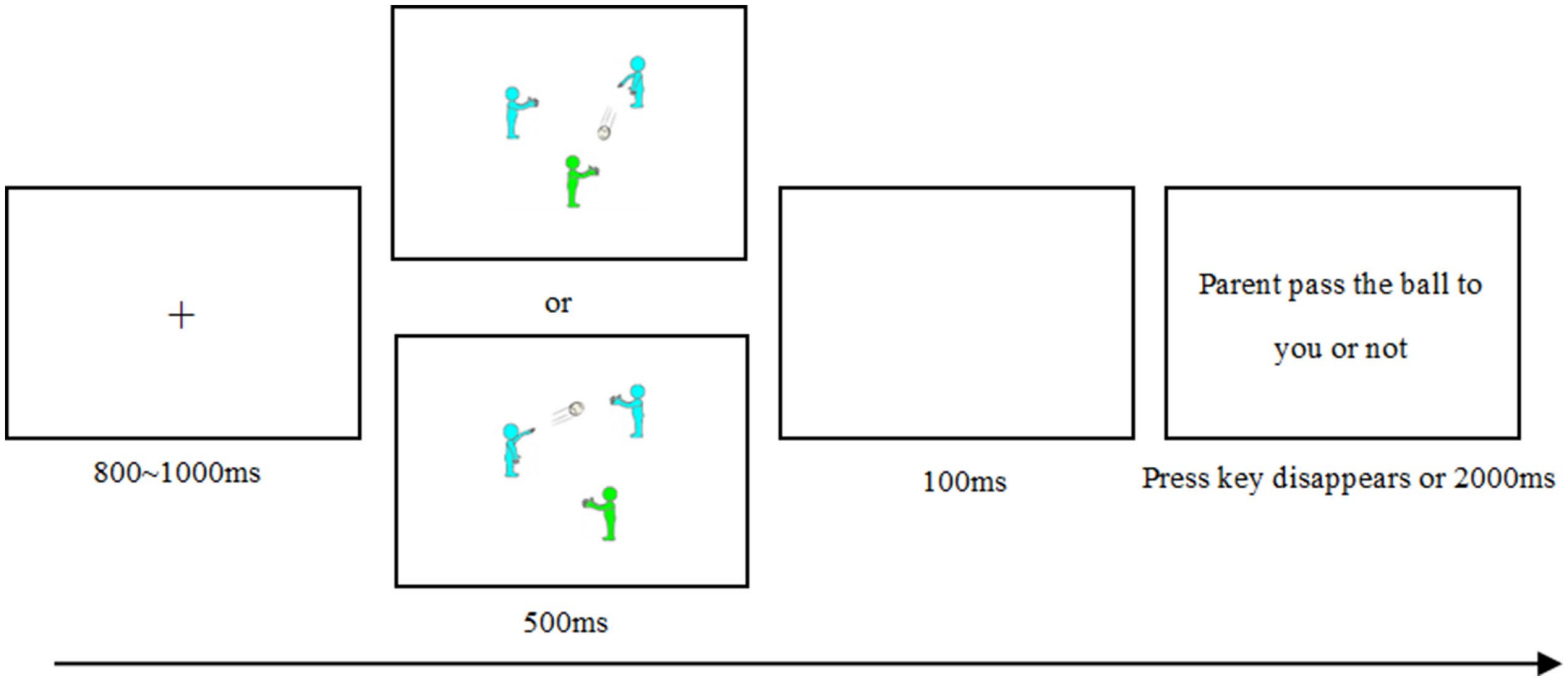
Schematic diagram of the experimental process of the passing ball task.

The specific process of each trial (see Figure 2) was as follows: ① The gaze point “+” appeared in the center of the screen to remind the participants to concentrate. ② Static passing ball pictures were presented randomly. At this time, participants did not do any key reaction and should immerse their imagination according to the picture situation. ③ Then, the participants were presented with a question “parent/friend/stranger/others pass the ball to you or not.” The participants responded on this screen by pressing the “D” key when parent/friend/stranger/others did not pass the ball to them, and pressing the “K” key when parent/friend/stranger/others did. Keys were balanced between participants.

Participants first completed 16 trials of exercises (eight times each of the ball passed to the participant and eight times when the ball was not passed to the participant) to familiarize themselves with the experimental process and operation. When the accuracy rate reached 95% or more, they entered the formal experiment. The formal experiment consisted of 80 trials, and the procedure was consistent with the exercise stage. The three exclusion groups initiated social exclusion by presenting 20% of the pictures, which passed the ball to the participants and 80% of not passing the ball to the participants. The others inclusion group initiated social inclusion by adopting two types of pictures with an equal probability of presentation.

In order to test the effectiveness of the entire activate process, a preliminary experiment was conducted before the formal study [participants information: 26 people in the parent exclusion group (14 males), M_age_ = 19.15 ± 0.88 years; 26 people in the friend exclusion group (12 males), M_age_ = 19.35 ± 0.80 years; 24 people in the stranger exclusion group (11 males), M_age_ = 18.92 ± 0.83 years; and 25 people in the others inclusion group (11 males), M_age_ = 19.40 ± 1.00 years]. Complete the post-cyberball questionnaire after the virtual pass ball task.

The post-cyberball questionnaire total score and each sub-dimension score measured after the pre-experiment were analyzed by between-subject variance analysis of the social priming group (parent exclusion, friend exclusion, stranger exclusion, and others inclusion). The main effects of the total score of the questionnaire [*F*(3, 97) = 16.63, *p* < 0.001, $\eta_{p}^{2}$ = 0.34] and the sub-dimensions such as sense of belonging [*F*(3, 97) = 19.88, *p* < 0.001, $\eta_{p}^{2}$ = 0.38], self-esteem [*F*(3, 97) = 10.98.63, *p* < 0.001, $\eta_{p}^{2}$ = 0.25], sense of control [*F*(3, 97) = 4.29, *p* = 0.007, $\eta_{p}^{2}$ = 0.12], and sense of meaning [*F*(3, 97) = 16.66, *p* < 0.001, $\eta_{p}^{2}$ = 0.34] were all significant. After the *post hoc* analysis, it was found that the scores of the social inclusion group in the Post-cyberball questionnaire and the sub-dimensions were significantly higher than the other three groups of social exclusion groups. This result proves that the whole experiment activate procedure was effective.

### 2.5. EEG data recording and analysis

The experimental EEG data was recorded using a 64-channel EEG recording system of Brian Product Company’s model Anti Champ. The horizontal EOG electrode was placed about 1 cm outside the lateral canthus of the right eye, and the vertical EOG electrode was placed about 1.5 cm in the lower eyelid of the left eye, and the forehead was grounded. The reference electrode on the line was Fz. The sampling frequency was 500 Hz, the filter bandpass was set to 0.01–100 Hz, and the resistance of each electrode point was reduced to less than 5 kΩ. Use Brain Vision Analyzer 2.2 to perform off-line analysis on the collected EEG data. First, preprocess the EEG data: re-reference, convert the recorded raw data into offline reference, and set the reference electrode to the average value of the electrodes at the bilateral mastoid; Filtering, using zero phase shift filtering, the parameters were low-pass 30 Hz and high-pass 0.1 Hz, removing power frequency 50 Hz interference; Independent component analysis (ICA) was used to eliminate eyeballs; Segmentation, the data were segmented according to the stimulus type. The segmentation time course was 1,000 ms, and the 200 ms before the stimulus presentation was selected as the baseline, and the 800 ms after the stimulus presentation was used as the analysis time course; After the baseline was corrected, the artifacts were removed, eliminate the trials where the absolute value of the peak was greater than 80 μV and the amplitude change per millisecond exceeds 50 μV; The trials under the four experimental conditions were superimposed and averaged, respectively. Combining the total average graph and existing researches, select P2 (220–300 ms), P3a (300–400 ms), and LPC (400–700 ms) components for statistical analysis of midline brain regions (frontal region: F1, Fz, F2; central region: FC1, FCz, FC2, C1, Cz, C2; parietal region: CP1, CPz, CP2, P1, Pz, P2; and occipital region: PO3, POz, PO4). The analysis software adopts SPSS19.0, and the *p* values of multiple comparisons after the analysis of variance were corrected by Bonferroni method.

## 3. Results

### 3.1. IOS measurement results

A between-subject ANOVA of the social priming group on intimacy measured by IOS found that the four groups of participants did not differ significantly in their ratings of intimacy with parents, friends, and strangers (*F*s < 1.64, *p*s > 0.186). Furthermore, the within-subject ANOVA was performed on the assessment of the intimacy of parents, friends, and strangers by participants in the four groups and found that, in each group, there were significant differences in intimacy ratings among parents, friends, and strangers (*F*s > 64.92, *p*s < 0.001). The above results show that the grouping of parents, friends, and strangers has a good gradient of intimacy.

### 3.2. ERP results

#### 3.2.1. P2

The ANOVA of the latency and peak of the P2 component in the midline brain regions (frontal, central, parietal, and occipital) found that, the P2 component peaks among the four groups were not significantly different in frontal [*F* (3, 85) = 0.83, *p* = 0.480] and central [*F* (3, 85) = 0.95, *p* = 0.421] regions. The peaks of the P2 components among the four groups were significantly different in the parietal region *F* (3, 85) = 2.89, *p* = 0.040, $\eta_{p}^{2}$ = 0.092. Combined with the *post hoc* analysis and descriptive results (others inclusion: 0.53 μV, parent exclusion: 0.26 μV, friend exclusion: 1.41 μV, stranger exclusion: 3.17 μV), it can be concluded that stranger exclusion induces larger peaks in P2 components than others inclusion (*p* = 0.021) and parent exclusion (*p* = 0.008) conditions. The peaks of the P2 components among the four groups were significantly different in the occipital region *F* (3, 85) = 3.71, *p* = 0.015, $\eta_{p}^{2}$ = 0.116. Combined with the *post hoc* analysis and descriptive results (others inclusion: 1.74 μV, parent exclusion: 1.75 μV, friend exclusion: 2.76 μV, and stranger exclusion: 4.86 μV), it can be inferred that stranger exclusion evoked a larger peak of P2 component than others inclusion (*p* = 0.006), parent exclusion (*p* = 0.004), and friend exclusion (*p* = 0.048). There were no significant differences in P2 component latency in all brain regions among the four groups (*F*s < 2.31, *p*s > 0.082). The P2 component peaks evoked by the exclusion group were significantly negatively correlated with the intimacy scores (*r* = −0.246, *p* = 0.042).

#### 3.2.2. P3a

The ANOVA of the social priming group on the peak and latency of the P3a component in the midline brain regions found that, the peaks of the P3a components among the four groups were marginally significantly in the frontal region *F* (3, 85) = 2.69, *p* = 0.051, $\eta_{p}^{2}$ = 0.087. Combined with the post-hoc analysis and descriptive results (others inclusion: −0.04 μV, parent exclusion: 1.04 μV, friend exclusion: 2.66 μV, and stranger exclusion: 3.10 μV), it can be inferred that stranger exclusion (*p* = 0.015) and friend exclusion (*p* = 0.032) induced larger peaks of P3a components than others inclusion condition. The evoked P3a component peaks were not significantly different in the central region among the four groups *F* (3, 85) = 1.73, *p* = 0.168. The evoked P3a component peaks were not significantly different in the parietal region among the four groups *F* (3, 85) = 2.52, *p* = 0.064. The peaks of the P3a components among the four groups were significantly different in the occipital region *F* (3, 85) = 6.53, *p* = 0.001, $\eta_{p}^{2}$ = 0.187. Combined with the *post hoc* analysis and descriptive results (others inclusion: 5.16 μV, parent exclusion: 6.12 μV, friend exclusion: 6.00 μV, and stranger exclusion: 9.24 μV), it can be inferred that stranger exclusion evoked larger peaks of P3a components than others inclusion (*p* < 0.001), parent exclusion (*p* = 0.002), and friend exclusion (*p* = 0.001). There were no significant differences in the latency of the P3a component among the four groups in all brain regions. The P3a component peaks evoked by the exclusion group were significantly negatively correlated with intimacy scores (*r* = −0.307, *p* = 0.010).

#### 3.2.3. LPC

ANOVA of social priming group on LPC component amplitudes in midline brain regions found that, the amplitudes of the LPC components among the four groups were significantly different in the frontal region *F* (3, 85) = 5.54, *p* = 0.002, $\eta_{p}^{2}$ = 0.164. Combined with the post-hoc analysis and descriptive results (others inclusion: −3.43 μV, parent exclusion: −2.13 μV, friend exclusion: −0.61 μV, and stranger exclusion: 0.83 μV), it can be inferred that the amplitude of LPC component induced by stranger exclusion was larger than others inclusion (*p* < 0.001) and parent exclusion (*p* = 0.007), and the amplitude of friend exclusion (*p* = 0.012) was larger than others inclusion. The amplitudes of the LPC components among the four groups were significantly different in the central region *F* (3, 85) = 6.99, *p* < 0.001, $\eta_{p}^{2}$ = 0.198. Combined with the post-hoc analysis and descriptive results (others inclusion: −1.23 μV, parent exclusion: −0.30 μV, friend exclusion: 1.41 μV, and stranger exclusion: 3.02 μV), it can be inferred that the amplitude of LPC component induced by stranger exclusion was larger than others inclusion (*p* < 0.001) and parent exclusion (*p* = 0.001), and the amplitude of friend exclusion (*p* = 0.009) was larger than others inclusion. The amplitudes of the LPC components among the four groups were significantly different in the parietal region *F* (3, 85) =8.10, *p* < 0.001, $\eta_{p}^{2}$ = 0.222. Combined with the *post hoc* analysis and descriptive results (others inclusion: 0.88 μV, parent exclusion: 1.89 μV, friend exclusion: 3.36 μV, and stranger exclusion: 5.50 μV), it can be inferred that the amplitude of the LPC component induced by stranger exclusion was larger than that of others inclusion (*p* < 0.001), parent exclusion (*p* < 0.001) and friend exclusion (*p* = 0.029), and the LPC composition induced by friend exclusion (*p* = 0.014) was larger than that of others inclusion. The amplitudes of the LPC components among the four groups were significantly different in the occipital region *F* (3, 85) =9.62, *p* < 0.001, $\eta_{p}^{2}$ = 0.253. Combined with the *post-hoc* analysis and descriptive results (others inclusion: −0.05 μV, parent exclusion: 1.87 μV, friend exclusion: 2.59 μV, and stranger exclusion: 4.48 μV), it can be inferred that the amplitude of LPC components induced by stranger exclusion was larger than that of others inclusion (*p* < 0.001), parent exclusion (*p* = 0.002)and friend exclusion (*p* = 0.023), parent exclusion (*p* = 0.026) and friend exclusion(*p* = 0.002) were larger than others inclusion. The amplitude of the LPC component evoked by the exclusion group was significantly negatively correlated with the intimacy scores (*r* = −0.305, *p* = 0.011).

## 4. Discussion

**Figure 3:**
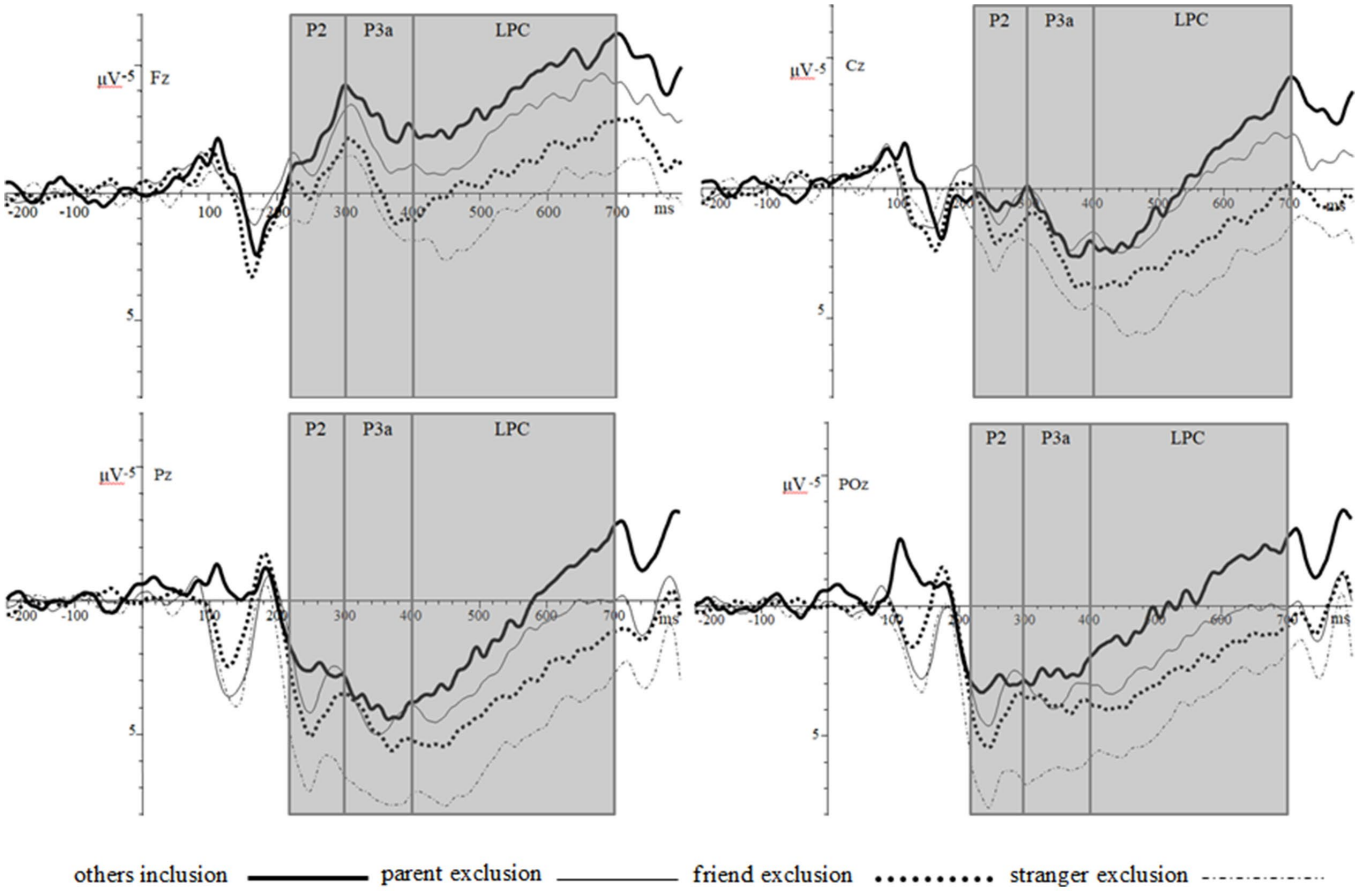
Total average ERPs waveforms of the four social priming groups.

**Figure 4:**
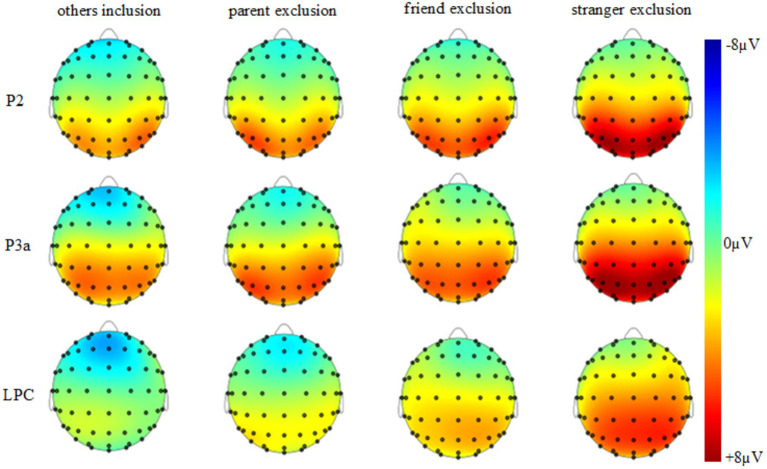
Topographic maps of the four social priming groups.

In this study, the static passing ball paradigm and combined with ERP technology were used to investigate the electrophysiological characteristics of individuals when they were excluded by people with different close and distant relationships. The results of this study showed that there was no significant difference in the peak of the P2 component between the four groups in the frontal and central regions; Stranger exclusion had a larger peak of P2 component than others inclusion and parent exclusion in the parietal region, and stranger exclusion had a larger peak of P2 components than others inclusion, parent exclusion, and friend exclusion conditions in the occipital region. In addition, combined with Figures 3, 4 and the descriptive results, it can be seen that in the parietal and occipital region, the peaks of P2 component induced by others inclusion, parent exclusion, friend exclusion, and stranger exclusion have a gradually increasing trend. The results of this study are basically consistent with those of previous electrophysiological studies of social exclusion. The results of this study are basically consistent with the results of previous electrophysiological studies on social exclusion. Previous studies have found that compared with social inclusion, social exclusion conditions induce larger amplitudes of early P2 (Key et al., 2005; Sreekrishnan et al., 2014) and N2 (Themanson et al., 2013; Weschke and Niedeggen, 2013) components. The present study further extends the findings on this basis: the P2 component peaks are relatively larger when individuals are excluded by the more distant relationship. Previous studies have shown that the P2 and N2 components mainly reflect the perception and processing of stimuli that require attention (Key et al., 2005; Sreekrishnan et al., 2014), and reflect the neural alertness activation of individual conflict monitoring in exclusion events (Themanson et al., 2013; Weschke and Niedeggen, 2013). Based on this and the results of this study, it can be inferred that a greater P2 component is induced when an individual is excluded, and when being excluded by the more distant relationship person, the induced P2 component peak is larger, indicating that the individual will be relatively more vigilant when facing the exclusion by the more distant relationship person. The reason for vigilance should be motivation. Some studies have found that when individuals are excluded, they will have the prevention-focused motivation mentioned in the regulatory focus theory, resulting in individuals showing higher alertness to potential threats (Molden et al., 2009; Park and Baumeister, 2015). It can be seen that the higher alertness to the distant relationship excluder indicates that the individual believes that the potential threat of being excluded by the more distant relationship person will be greater.

After the awareness of exclusion, there is an evaluation stage of exclusion (Kawamoto et al., 2015), and the evaluation results are closely related to the social pain and the behavioral methods used to cope with it. Previous studies using the passing ball paradigm have shown that individuals induce two subcomponents of the P3 family, P3a and P3b, when they are excluded. For example, studies have shown that when individuals are excluded, they can cause negative emotions in participants, which is manifested as greater amplitude of the P3a component evoked in the frontal–parietal region, and the activation of this component is related to the activation of the anterior cingulate cortex (ACC; Gutz et al., 2011; Weschke and Niedeggen, 2013, 2016). The results of this study are basically consistent with those of previous studies and have been expanded. This study found that the peak of P3a component induced by stranger exclusion in the frontal region was larger than that of others inclusion and parent exclusion. In addition, combined with Figures 3, 4 and the descriptive results, it can be seen that the P3a component peaks induced by others inclusion, parent exclusion, friend exclusion, and stranger exclusion have a gradually increasing trend in the frontal region. Correlative neuroimaging studies reveal that activation of the anterior cingulate cortex (ACC) is associated with negative emotions and pain responses to social exclusion (Gutz et al., 2011; Weschke and Niedeggen, 2015). It can be inferred that individuals will have negative emotions and social pain when faced with exclusion, and may have more negative emotional and pain responses when being excluded by the more distant relationship person.

Another P3b component evoked during the evaluation phase in studies related to social exclusion is related to attention, evaluation, and classification of stimuli (Gutz et al., 2011; Weschke and Niedeggen, 2013, 2015; Kiat et al., 2017; Kiat and Cheadle, 2017). In this study, the static passing ball paradigm with identity information was adopted. According to the waveforms in Figure 3, experiments in this paradigm induced LPC components that expressed the same meaning—reflecting attention and evaluation of stimuli (Citron, 2012). A large number of previous studies have shown that compared with social inclusion conditions, the P3b component induced by social exclusion conditions has a larger amplitude (Crowley et al., 2010; Themanson et al., 2013; Weschke and Niedeggen, 2013, 2016; Gutz et al., 2015). The results of this study showed that the amplitudes of LPC components evoked in the midline brain regions (frontal, central, parietal, and occipital) were greater under the condition of stranger exclusion. In addition, combined with Figures 3, 4 and the descriptive results, it can be seen that the amplitude of the LPC component induced by the midline brain region from others inclusion, parent exclusion, and friend exclusion to stranger exclusion has a gradually increasing trend. Thus, this study not only fully supports the conclusion that the amplitude of related electrophysiological components induced by social exclusion is larger than that induced by social inclusion from the multiple dimensions, but also further finds that the amplitudes of the relevant electrophysiological components evoked are relatively larger when the individual is excluded by the more distant relationship person. Previous studies have shown that the increase in the amplitude of the P3b component not only indicates that individuals will pay more attention to exclusion information, but also reflects the intensity of exclusion perceived by individuals (Gutz et al., 2011; Kawamoto and Nittono, 2013; Themanson et al., 2015). Based on this and combined with the results of this study, it can be seen that individuals will experience a sense of exclusion when they are excluded by people with different close and distant relationships, and the perceived exclusion experience will be stronger when they are excluded by those who are more distant relationship. In a related study, it was also pointed out that the P3b component was strongly associated with self-reported social pain experience after social exclusion (Crowley et al., 2010; Themanson et al., 2013). Therefore, it can be speculated that the social pain experience experienced by individuals who are excluded by the more alienated person will be stronger. However, this speculation needs further research to be demonstrated.

Previous studies that explored the electrophysiological components evoked by self-related close and distant relationship information found that the electrophysiological components (P2 and P3) evoked by self-information had the largest amplitudes (Tacikowski and Nowicka, 2010; Fan et al., 2013). However, the conclusions of close and distant relationship information are not uniform, and some studies have found that there is no significant difference in the electrophysiological components induced by close and distant relationship information (such as names of celebrities and strangers; Tacikowski and Nowicka, 2010). Some studies have also pointed out that there is a degree effect of electrophysiological components induced by close and distant relationship information, such as the name of acquaintances, which induces larger P3 amplitude than the name of strangers (Fan et al., 2013). This study found that the amplitude of the electrophysiological component induced by stranger exclusion was the largest, and the degree effect was just opposite to that in the study of close and distant relationship information. It can be concluded that the electrophysiological components induced by the individual’s familiarity with the close and distant relationship information do not affect the results of this study, but can prove the reliability and scientificity of the results of this study from another perspective. In addition, there was a significant negative correlation between intimacy scores and the electrophysiological components induced by different close and distant relationship excluders, which further proved that the differences in electrophysiological components could reflect the processing of excluders with different close and distant relationships. Therefore, the above results of P2, P3a, and LPC components indicate that the cognitive processing pattern of individuals when they are excluded by someone who is more distant relationship is as follows: In the face of the exclusion by the more distant relationship people, individuals will first be relatively more alert, and then produce more negative emotions, so as to perceive the stronger exclusion experience. In addition, the results of the three components are also helpful to understand the physiological reasons behind the behavioral choice of individuals to cope with exclusion mentioned in the multivariate motivation model, and reveal the electrophysiological basis behind the different coping behaviors of individuals in the face of exclusion by different close and distant relationships. For example, the results of this study well explain the reasons and physiological basis behind previous findings that individuals will adopt more negative behaviors such as avoidance when coping with exclusion from strangers than when coping with exclusion from close ones (Uskul and Over, 2014).

## 5. Conclusion

This study used the static passing ball paradigm incorporating identity information and combined with ERP technology to investigate the electrophysiological characteristics of individuals when they were excluded by people with different close and distant relationships. The study found: (1) Greater P2, P3a, and LPC components are induced when an individual is excluded than when included; (2) The P2, P3a, and LPC components induced by individuals who are excluded by people with different close and distant relationships have a degree effect, which are manifested that the amplitudes of the induced P2, P3a, and LPC components are relatively larger when they are excluded by the more distant relationship people. The above results indicate that individuals experience exclusion when they are excluded by people with different close and distant relationships, and become more alert and perceive a stronger experience of exclusion when they are excluded by people who are more distant relationship.

## Data availability statement

The raw data supporting the conclusions of this article will be made available by the authors, without undue reservation.

## Ethics statement

The studies involving human participants were reviewed and approved by School of Education, Zhejiang International Studies University. The patients/participants provided their written informed consent to participate in this study.

## Author contributions

PZ, MZ, and XG contributed to conception and design of the study. JH organized the database. PZ performed the statistical analysis and wrote the first draft of the manuscript. All authors contributed to the article and approved the submitted version.

## Funding

This work was supported by the Philosophy and social science planning project of Zhejiang Province (22NDQN265YB), Youth Fund Project for Humanities and Social Science Research of the Ministry of Education of China (22YJCZH253), Educational Science Planning project of Zhejiang Province (2021SCG340), and Boda Teacher Scientific Research Promotion Special project of Zhejiang International Studies University (2021QNYB2).

## Conflict of interest

The authors declare that the research was conducted in the absence of any commercial or financial relationships that could be construed as a potential conflict of interest.

## Publisher’s note

All claims expressed in this article are solely those of the authors and do not necessarily represent those of their affiliated organizations, or those of the publisher, the editors and the reviewers. Any product that may be evaluated in this article, or claim that may be made by its manufacturer, is not guaranteed or endorsed by the publisher.
